# Risk of COVID-19 transmission in heterogeneous age groups and effective vaccination strategy in Korea: a mathematical modeling study

**DOI:** 10.4178/epih.e2021059

**Published:** 2021-09-08

**Authors:** Youngsuk Ko, Jacob Lee, Yubin Seo, Eunok Jung

**Affiliations:** 1 Department of Mathematics, Konkuk University, Seoul, Korea; 2 Division of Infectious Disease, Department of Internal Medicine, Kangnam Sacred Heart Hospital, Hallym University College of Medicine, Seoul, Korea

**Keywords:** COVID-19, Theoretical models, Vaccination, Physical distancing, Republic of Korea

## Abstract

**OBJECTIVES:**

This study aims to analyze the possibility and conditions of maintaining an effective reproductive number below 1 using a mathematical model.

**METHODS:**

The total population was divided into five age groups (0-17, 18-29, 30-59, 60-74, and ≥75 years). Maximum likelihood estimation (MLE) was used to estimate the transmission rate of each age group. Mathematical model simulation was conducted until December 31, 2021, by establishing various strategies for vaccination and social distancing without considering variants.

**RESULTS:**

MLE results revealed that the group aged 0-17 years had a lower risk of transmission than other age groups, and the older age group had relatively high risks of infection. If 70% of the population will be vaccinated by the end of 2021, then simulations showed that even if social distancing was eased, the effective reproductive number would remain below 1 near August if it was not at the level of the third re-spreading period. However, if social distancing was eased and it reached the level of the re-spreading period, the effective reproductive number could be below 1 at the end of 2021.

**CONCLUSIONS:**

Considering both stable and worsened situations, simulation results emphasized that sufficient vaccine supply and control of the epidemic by maintaining social distancing to prevent an outbreak at the level of the re-spreading period are necessary to minimize mortality and maintain the effective reproductive number below 1.

## INTRODUCTION

Coronavirus disease 2019 (COVID-19), which started in China at the end of 2019, has spread worldwide, and 160 million confirmed cases, 3 million deaths, and 600,000 daily new confirmed cases have been reported as of May 25, 2021. The high-risk of the elderly population of having severe illness due to COVID-19 burdens the medical system worldwide, and non-pharmacological interventions such as lockdown cause social and economic burdens [1]. The AZD1222 vaccine was first approved for emergency use authorization in the United Kingdom in December 2020, and BNT162 has additionally been approved and administered in several countries [1,2].

On February 16, 2020, the first community infection occurred in Korea. The first epidemic wave started in Daegu/Gyeongbuk following a mass infection in the religious groups. Since then, Korea has experienced its second epidemic wave centered around the metropolitan area and third epidemic wave, which was caused by sporadic infections [3-5]. After the maximum number of confirmed cases in the third wave, social distancing was strengthened, and a policy banning private gatherings of five or more people was implemented in the metropolitan area.

Vaccination in Korea started for the first time on February 26, 2021, and as of May 25 2021, a total of 3,864,784 people has received at least one dose [6]. The Korean government plans to administer 190 million doses of the vaccine by the end of 2021. In order to minimize the number of severely ill patients and deaths, vaccinations are scheduled for the elderly in the second quarter, starting with residents and workers of long-term care hospitals and nursing facilities and employees at high-risk medical institutions [7,8]. According to the press release dated May 6, 2021 of the Korea Disease Control and Prevention Agency (KDCA), the effectiveness of vaccines was found to be 86% for AZD1222 and 89.7% for BNT162 two weeks after the first dose for those aged ≥ 60 years, and the adverse reaction reporting rate was 0.1% [8].

Mathematical models have been developed to study the spread of COVID-19 in Korea even before the first epidemic wave. When COVID-19 was only prevalent in China in the early 2020s, a study was conducted to investigate the scale of an outbreak once the disease arrives in Korea. The model was used to interpret initial epidemic situation considering changes in the population behavior. Another study was conducted to analyze the effect of social distancing using mobility data observed during the first epidemic wave [9-11].

Motivated by earlier studies, this study aims to formulate a mathematical model that considers age groups and vaccination. The period for the model simulation is from October 12, 2020, to May 25, 2021, and the extended simulation includes the vaccination plan for 2021. Considering factors such as social distancing, amount of vaccination, and vaccination method, this study aims to examine the possibility of maintaining the effective reproductive number below 1 and design strategies to minimize mortality.

## MATERIALS AND METHODS

### Estimation of transmission rate matrix between age groups using maximum likelihood estimation

Age, presence of symptoms, symptom onset date, and diagnosis date of individual confirmed cases from October 12, 2020, to February 14, 2021, provided by the KDCA were used to estimate transmission rates between age groups using the maximum likelihood estimation (MLE) method. The total population was divided into five groups considering vaccination policy and behavioral heterogeneity, and accordingly each age group and population were as follows [12]:

I: 0-17 years (7,625,814 people); II: 18-29 years (7,781,166 people); III: 30-59 years (23,650,843 people); IV: 60-74 years (9,020,169 people); V: ≥ 75 years (3,624,108 people).

The transmission rates between age groups form a 5× 5 matrix. It is assumed that the transmission rate matrix is symmetric, and the number of estimated transmission rates is 15. To establish the MLE, we assume a homogenous mixing of infectors and infectees in the community and an exponential distribution for the event of infection. Hence, if the rate at which an individual in age group *Y* infects an individual in age group *X* is β*_XY_*, then the probability that individual *i* in age group *X* at time *t* is not infected at time *t*+1 is *p_sur,X,i_*(*t*)= $\exp(-\frac{\Sigma \beta_{XY}I_{y}(t)}{N})$ [13]. Here, *I_Y_*(*t*) is the number of infected people in age group Y that can transmit the disease at time *t*, and *N* is the total population. Conversely, the probability of being infected at time *t*+1 is *p_inf,X,i_*(*t*)= $1-\exp(-\frac{\Sigma \beta_{XY}I_{y}(t)}{N})$ . The likelihood of all individuals (including those not infected until the last moment) can be formulated using the probability of being not infected and infected, which is as follows:

$$L=\prod_{X}^{}\{\prod_{i\in \Lambda_{I,X}}^{}[(\prod_{j=0}^{t_{inf,x,i}-2}p_{sur,X,j}(t))p_{inf,X,i}(t_{inf,X,i}-1)]\prod_{k\in \Lambda_{S,X}}^{}(\prod_{j=0}^{t_{f}-1}p_{sur,X,j}(t))\}$$

Since there was no information on the date when individual patients were infected, symptomatic patients are assumed to be infected four days before symptom onset and can transmit the virus two days before symptom onset until one day before diagnosis [14,15]. The periods of infection transmission for asymptomatic infected patients were set in the same way as those with symptoms using the average value of symptomatic patients.

### Mathematical modeling for the COVID-19 pandemic

**Figure 1.**
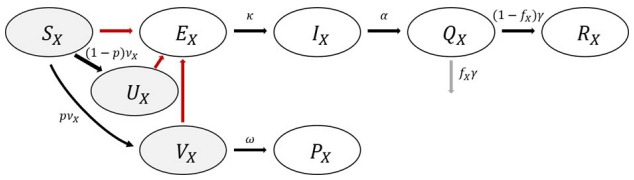
Flowchart of coronavirus disease 2019 (COVID-19) mathematical model considering age group and vaccination.

Based on the five age groups considered earlier, a mathematical model reflecting vaccination was developed. According to the epidemiological characteristics of COVID-19, the total population was divided into Susceptible (*S*), Latent or Exposed (*E*), Infectious (*I*), Isolated (*Q*), and Recovered (*R*) groups. Three additional groups (*U, V, P*) were considered to reflect vaccination: the group who is vaccinated but not effectively (Unprotected: *U*), the group that had been effectively vaccinated but not yet immunized (Vaccinated: *V*), and the group that was effectively vaccinated and became immunized after a certain period (Protected: *P*). Therefore, the groups *S, U*, and *V* can be infected, and it was assumed that all had the same susceptibility. The mathematical model flowchart is shown in Figure 1, and the governing differential equations are as follows.

$$\frac{dS_{X}}{dt}=-\Lambda_{X}S_{X}-v_{x},\;\frac{dE_{X}}{dt}=\Lambda_{X}(S_{X}+U_{X}+V_{X})-kE_{X},\;\frac{dI_{X}}{dt}=kE_{X}-aI_{X},\;\frac{dQ_{X}}{dt}=aI_{X}-\gamma Q_{X},\;\frac{dR_{X}}{dt}=\gamma (1-f_{x})Q_{X},\;\frac{dV_{X}}{dt}=(1-p)v_{X}-\Lambda_{X}U_{X},\;\frac{dV_{X}}{dt}=pv_{X}-\Lambda_{X}V_{X}-\omega V_{X},\;\frac{dP_{X}}{dt}=\omega V_{X},\;\Lambda_{X}=\beta_{0}(t)\frac{\Sigma \beta_{XY}I_{Y}}{N},\;N=\Sigma S_{X}+E_{X}+I_{X}+R_{X}+U_{X}+V_{X}+P_{X}$$

In the model, the subscript *X* denotes the age group, and β*_XY_* was the estimated transmission rate between age groups using MLE, which represented the average transmission among age groups during the third wave of the epidemic. It was assumed that the transmission rates between age groups were the same during the entire simulation period. However, epidemic trends change depending on intervention policies and transmission patterns, so an adjusting constant, β_0_(*t*), was considered to the force of infection to account for intervention policy and transmission pattern that changed every period. Vaccination was reflected in the model using νX, and the actual amount of vaccination was reflected until the estimated simulation period of β_0_(*t*) [6]. The phase dependent parameter β_0_(*t*) was estimated by mini mizing the square error of the number of cumulative confirmed cases in the model (*∫∑αI_X_ dt*) and the reported data. The period of estimation was from October 12, 2020, the start of social distancing phase 1 before the third wave in Korea, to May 25, 2021 [16]. The parameter, *p*, represents the average vaccine effectiveness. The amount of vaccination and vaccine effectiveness of each type of vaccine administered in Korea were calculated as a weighted average and set to 0.84 [17]. The values of the model parameters are listed in Table 1, and the mortality rate by age group was calculated based on the data of individual confirmed cases who were released from isolation. The effective reproductive number R(*t*) calculated using the next-generation method was presented in the results section [18].

### 2021 Vaccination scenarios

In order to predict the situation of the COVID-19 epidemic following vaccination, scenarios were considered based on the phase transition criteria of the social distancing system reorganization plan of the Ministry of Health and Welfare [21]. Under the reorganization plan, the social distancing phase is determined by the weekly average number of new confirmed cases per 100,000 population, considering the availability of regional intensive care units. At the national level, the transition from phase 1 to phase 2 requires that the weekly average number of new confirmed cases per 100,000 population is 363. To transition to phases 3 and 4, the weekly average number of new confirmed cases per 100,000 population are 778 and 1,556 people, respectively. Phase 3 was not declared until May 25, 2021, since the weekly average of confirmed cases was below 600. Scenarios were designed by assuming that phases 1 and 2 would continue during the second half of 2021 without the emergence of variants, re-spreading, or eradication of the COVID-19 epidemic. In both scenarios, the obtained transmission rate matrix and estimated values of adjustment factor during the third epidemic wave β_0_(*t*) were used.

■ Scenario 1 (S1): Phase 1 is applied with the largest value of β_0_(*t*) during the third wave (when R(*t*)= 1.68).
■ Scenario 2 (S2): Phase 1 is applied with the value of β_0_(*t*) during the period right before the third wave (when R(*t*)= 1.22, October 12 to November 3, 2020).

Note that phase 2 is applied with the smallest value of β_0_(*t*) during the third wave both in scenarios 1 and 2 (when R(*t*)= 0.71).

Three vaccination strategies were considered; priority vaccination for the elderly group (in the order of group V-IV-III-II), simultaneous vaccination with the same proportion of all age groups except for those under aged 17 (I), and no vaccination. In the priority vaccination strategy, the next age group beginning from the oldest, was vaccinated after all the individuals in their age group were given the full dose of vaccine. In both priority and simultaneous vaccination strategies, individuals in age group I are not vaccinated. Since the total amount of vaccination may change flexibly depending on the situation, the target proportion of the population to be vaccinated by December 31, 2021 is set to 60%, 70%, or 80% (31,021,260, 36,191,470, and 41,361,680) of the total population of Korea. This means that if 60% (70 to 80%) of the total population is vaccinated, approximately 70% (82 to 94%) of the remaining age groups except for the age group I will be vaccinated. Accordingly, the total number of simulations is 14. The simulation period for the scenarios begins from May 25, 2021, the last day of parameter estimation, to December 31, 2021, and the daily vaccination dose is given equally, on average, according to the set total vaccination dose. The observed figures are the number of additional daily new confirmed cases since May 25, 2021, cumulative confirmed cases and deaths, effective reproductive number, and time when the effective reproductive number is consistently below 1 (when the effective reproductive number is below 1 even if the level of social distancing is eased).

## RESULTS

### Estimation of transmission rate matrix between age groups using maximum likelihood estimation

**Figure 2.**
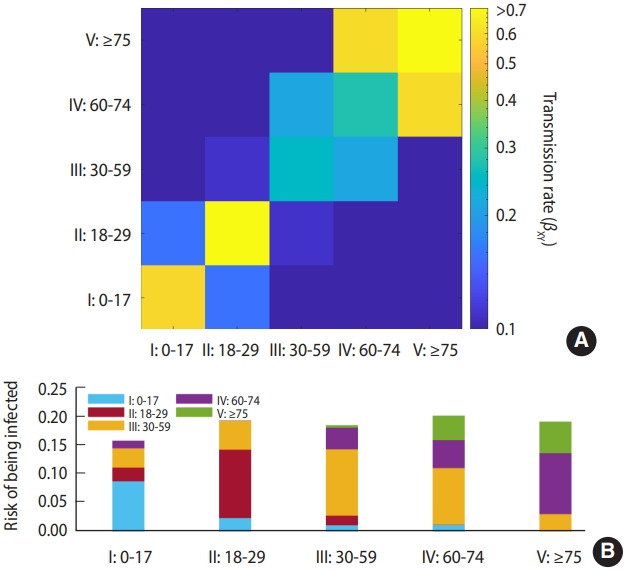
Maximum likelihood estimation results. (A) Estimated transmission rates (log-scaled). (B) Population-adjusted risk of being infected, which is calculated by taking elementwise product of row-vectored population size of age groups and transmission rate matrix and dividing all entries by total population size.

Estimated transmission rate between age groups (β*_XY_*) is visualized in Figure 2. Figure 2A is the value of the log-scaled transmission rates, and Figure 2B is the result of calculating the risk of being infected adjusted according to the population of each age group. The entries of the matrix are listed in Supplementary Material 1. The transmission rate was estimated to be high in the diagonal components; the highest was for the group aged 18-29 years (II) at 0.79, and the second highest was for the group aged ≥ 75 years (V) at 0.78.

Figure 2B displays the group dependent risk of exposure to infection using the adjusted population. This risk was calculated by multiplying the sum of the column elements by the value obtained after dividing the population in the age group corresponding to each column by the total population. This relative risk of infection is in the order of IV-II-V-III-I, from the highest. The group aged 60-74 years (IV) had the highest risk of being infected by other groups at 0.15, and among them, the infection risk by the group aged 30-59 years (III) accounted for more than half with 0.09. The age groups from 0 years to 59 years (I, II, III) had the lowest risk of infection by other groups at 0.07. The risk of infection among the same age group was the highest at 0.12 in the group aged 18-29 years (II) and the lowest at 0.05 in those aged 60-74 years (IV).

### Analysis of the effective reproductive number and the ratio of confirmed cases by age group

**Figure 3.**
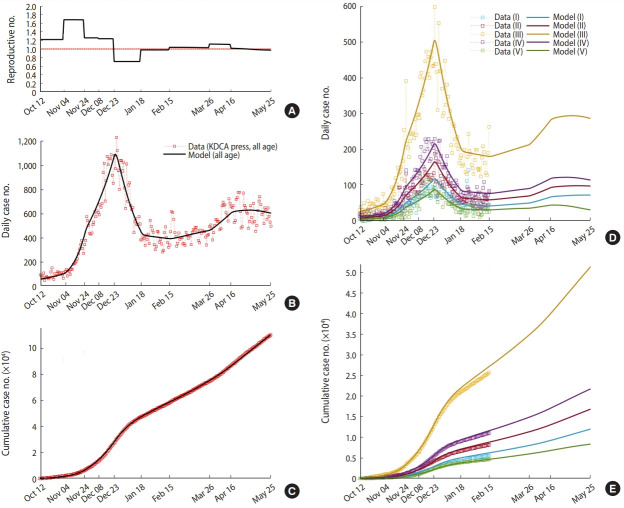
Data-fitting results (A) effective reproductive number, (B) daily cases (all age), (C) cumulative cases (all age), (D) daily cases of the five age groups, and (E) cumulative cases of the five age groups. In (B) and (C), the red squares depict data aggregated from the KDCA daily presses and the black solid curves are data-fitted model simulation result in all age. In (D) and (E), the colored squares depict data aggregated from the epidemiological records of individual coronavirus disease 2019 (COVID-19) cases and the colored solid curves are model simulation results of the five age groups. KDCA, Korea Disease Control and Prevention Agency.

Figure 3 shows the estimation results of the adjusting constant, β_0_(*t*), according to the government’s intervention policy. Figure 3A is the effective reproductive number, Figure 3 are the mathematical model simulation results (dark curve) and the KDCA press release data (red squares) for the number of daily and cumulative confirmed cases for all age groups, respectively. The graphs comparing the number of confirmed cases by age group and the model simulation results are shown in Figure 3D and E.

The adjusting constant, β_0_(*t*), was estimated to be 1.09 (effective reproductive number of 1.22) between October 12 and November 3, 2020, when phase 1 of social distancing was implemented at the start time of the model, and it was estimated to be the highest at 1.50 (effective reproductive number of 1.68) between November 4 and November 24, 2020. It was estimated to be the smallest at 0.64 (effective reproductive number of 0.71) between December 23, 2020 and January 18, 2021, when the social distancing was strengthened to phase 2.5, and when the ban on private gatherings with five or more people began in the metropolitan area.

**Figure 4.**
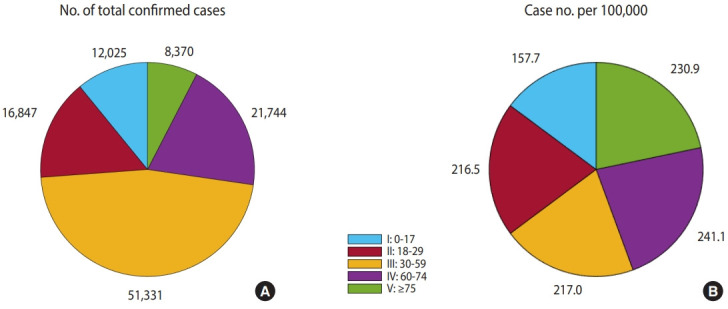
Model results in May 25, 2021 (A) confirmed case number and (B) case number per 100,000 of each age group.

Figure 4 shows mathematical model simulation results for each age group until May 25, 2021. In Figure 4A, the group aged 30-59 years (III) accounts for the largest number (n= 51,331) of the total number of confirmed cases. Figure 4B shows the number of confirmed cases per 100,000 people, adjusted with the number of people in each age group. It was observed that the group aged 0-17 years (I) had less cases than other age groups (157.7). The number of cases for the remaining age groups was around 200, of which the group aged 60-74 years (IV) showed the highest number at 241.1.

### 2021 Vaccination scenario simulation results

**Figure 5.**
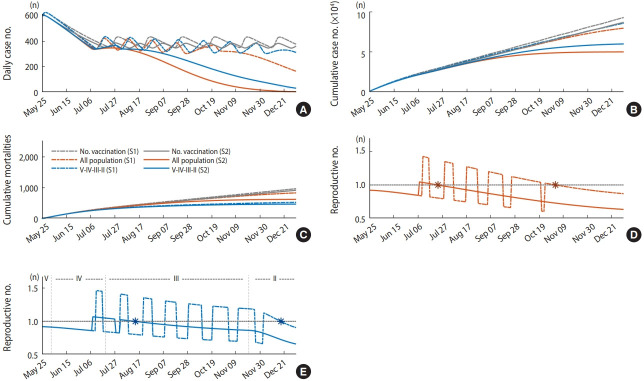
Extended model simulation results when 70% of the population except those aged under 18 years (36,191,470) is vaccinated (A) and (B) displays the number of daily and cumulative cases, respectively, (C) shows the number of additional mortalities, and (D) and (E) represents the effective reproductive number for scenario 1 and 2, respectively (the asterisk marker indicates the timing that reproductive number is consistently below 1). Note that text on the figure in panel (E) indicates which age group is vaccinated in each phase.

Figure 5 shows the simulation results for the scenarios when 70% of the total population (approximately 140,000 vaccination per day) is vaccinated by December 31, 2021. The colors of the graph indicate the vaccination strategies. The gray curve shows the case when there is no vaccination, the red curve indicates that all age groups are vaccinated at the same rate, and the blue curve indicates that the older age group is vaccinated first. In each strategy, the solid and the dotted lines describe scenarios where β_0_(*t*) is varied according to the social distancing level. The dotted line is the simulation result of scenario 1, and the solid line is scenario 2. If there is a point when the effective reproductive number is consistently below 1, the time is marked with an asterisk. The results of vaccinating 60% and 80% of the total population and varying the vaccine effectiveness to 65%, 70%, 75%, 80%, and 90% are summarized in Supplementary Material 2.

Table 2 summarizes the time when the reproductive number reaches consistently below 1, the number of additional confirmed cases, and the additional mortality number for each scenario. It was observed that the case when the total population was simultaneously vaccinated with equal rates, except for the group aged under 17, had fewer confirmed cases than the elderly priority vaccination strategy. If 80% of the total population was vaccinated at the same rate, the number of additional confirmed cases was 44,859, the smallest among all scenarios. It was identified that in all scenarios, except for the case where there was the highest number of deaths among the elderly priority vaccination scenarios (n= 588), the number of deaths was lower than the case with the lowest number of deaths (n= 581) among the scenarios when vaccination was given simultaneously to all age groups except group I. It was observed that the time when the effective reproductive number reaches consistently below 1 is achieved earlier when the vaccination rate increases, and it was reached faster when the total population is vaccinated at the same rate. Simulation results show that as in scenario 2, even if social distancing is eased, in a situation where the level of re-spreading does not reached, The time when the effective reproductive reaches below 1 may start around mid-July if the total population is vaccinated equally (Figure 5D solid curve), or mid-August if the elderly are vaccinated first (Figure 5E solid curve).

## DISCUSSION

Estimation of the transmission rate between age groups using MLE showed a high transmission rate among the group aged 18-29 years and in the group aged ≥ 60 years (Figure 2). The result adjusted by the population showed that the risk of spreading the disease in the third wave of the epidemic was not limited to a specific age group but was observed in all age groups. The group aged 0-17 years showed a relatively low transmission rate compared to other age groups, not only due to the effect of school closures, delay of school opening, and online lessons, which are part of social distancing, but also due to the nature of the virus itself, in the sense that the transmission rate of the virus among children and adolescents is lower than that of adults [22]. The school-age group had an estimated nine times less transmission rate than the rest of the age groups [23].

The results of the extended model simulation from May 25, 2021 extended to December 31, 2021, show that maintaining social distancing is crucial for an effective vaccination strategy. Scenarios S1 and S2 were established considering different vaccination strategies (simultaneous vaccination for all age groups and priority vaccination for the elderly) and social distancing situations (considering whether the transmission rate would increase to the level of a re-spreading of third wave (S1, at the time when R(*t*)= 1.68), or whether it would be similar to phase 1 of distancing implemented on October 12, 2020 (S2, at the time when R(*t*)= 1.22)). In S1, with the elderly having vaccination priority, even if 70% of the population is vaccinated during 2021, it is not until December that the effective reproductive number can start to be consistently below 1. This is not, however, conclude that priority vaccination for the elderly is not appropriate. Rather, when the elderly is vaccinated on priority, mortality number is always lower than of the scenarios when the vaccine is administered in equal proportion to the total population, excluding 588 people in the worst-case scenario (vaccination rate of 60%, scenario 1) (Table 2). Simulation results showed that if social distancing is maintained as in S2, it is possible that the effective reproductive number begins to remain consistently below 1 in August when 70% of the population is vaccinated by the end of December (Figure 5D and E solid lines).

This study had some limitations. First, to reduce the complexity of the model, second dose vaccination was not considered, and the type of vaccine and vaccine effectiveness were reflected on an average. In addition, waning immunity to vaccines and having less probability of being infected due to vaccination were not considered. Second, the transmission rate between age groups is likely to have changed due to vaccination that has begun recently, and in particular, the transmission rate in the elderly group may have been overestimated in this study. However, this study reflected the overall average transmission during the third wave of the epidemic. Due to data limitations, the estimated transmission rate, assuming homogeneous mixing in the community, does not imply the causal relationship of the actual infection. Hence, it may differ from the actual contact trend. Third, this study did not reflect the increase in transmission due to the appearance of variants. Hence, reaching a consistent effective reproductive number of below 1 may be delayed in each scenario due to the emergence of variants. However, in the eased social distancing scenario of this study, the high transmission rate (at the time of R(*t*)= 1.68) can also be interpreted as one reflecting the increase in transmission caused by variants. Further studies will be conducted considering second dose vaccination, transmission rate by epidemic period, waning immunity, detailed vaccine effectiveness, and variants.

In conclusion, this study developed a mathematical model where the transmission rates between age groups are estimated using MLE based on Korean data and analyzed the results in terms of effective reproductive number, the number of infected people, and minimizing mortality number by simulating various scenarios considering the 2021 vaccination plan. A critical result of this study is that effective vaccination strategies should be combined with vaccination priority and rapid vaccination as well as social distancing to prevent the effective reproductive number from reaching the level of re-spreading of third wave.

The priority vaccination strategy for the elderly significantly reduces the mortality number, indicating that the purpose and effect of currently established vaccination priorities are the same. However, in the worsening situation, simulation results show that the epidemic will continue in 2021, and even in such situations, the effectiveness of minimizing the mortality number remains unchanged. Thus, the results of the study emphasize the importance of social distancing rather than changing vaccination priorities. It is identified through mathematical modeling, that if 70% of the population is vaccinated by the end of December 2021 and social distancing is implemented to prevent the level of re-spreading during the third wave, the effective reproductive number may continue to be below 1 from around August 2021.

## Figures and Tables

**Table 1.** Model parameters

Symbol	Description	Value	Reference
1/*κ*	Latent period (d)	2.1	[14,15]
1/*α*	Average duration from infectious to isolated (d)	6	[15,19]
1/*γ*	Average isolated duration (d)	20	[20]
f_*X*_	Case fatality rate (%) of age group *X*	0.00 (I: 0-17)	
		0.02 (II: 18-29)	
		0.19 (III: 30-59)	
		1.56 (IV: 60-74)	
		14.18 (V: ≥75)	
1/*ω*	Average duration from vaccinated to protected (d)	14	[17]
*p*	Vaccine effectiveness (%)	84	[17]
*β*_0_(*t*)	Transmission rate adjusted constant	1.09 (Oct 12 to Nov 3)	Data-fitted
		1.50 (Nov 4 to Nov 23)	
		1.13 (Nov 24 to Dec 7)	
		1.11 (Dec 8 to Dec 22)	
		0.64 (Dec 23 to Jan 17)	
		0.88 (Jan 18 to Feb 14)	
		0.93 (Feb 15 to Mar 25)	
		1.01 (Mar 26 to Apr 15)	
		0.93 (Apr 16 to May 25)	

**Table 2.** Extended model simulation results considering various scenarios

Variables	Timing that reproductive no. is consistently below 1			Additional incidence no. (decreasing rate, %)			Additional mortality no. (decreasing rate, %)		
No vaccination									
S1	-			92,939			978		
S2	-			86,906			926		
Proportion of vaccinated (% of population)	60	70	80	60	70	80	60	70	80
All population									
S1	Nov 30	Nov 2	Oct 14	84,609 (9.0)	79,598 (14.3)	73,654 (20.7)	904 (7.6)	842 (13.9)	777 (20.1)
S2	Aug 01	Jul 23	Jul 17	55,260 (36.4)	49,851 (42.6)	44,859 (48.4)	689 (25.6)	632 (31.8)	581 (37.3)
Priority to the elderly									
S1	Dec 26	Dec 18	Dec 14	86,269 (7.2)	85,905 (7.6)	84,833 (8.7)	588 (39.8)	534 (45.3)	487 (50.2)
S2	Aug 22	Aug 14	Aug 08	64,778 (25.5)	75,673 (31.2)	57,359 (34.0)	526 (43.2)	477 (48.5)	442 (52.3)

S1, scenario 1: phase 1 is applied with the largest value of *β*_0_(*t*) during the third wave (when *R*(*t*)=1.68); S2, scenario 2: phase 1 is applied with the value of *β*_0_(*t*) during the period right before the third wave (when *R*(*t*)=1.22, October 12 to November 3, 2020).

## SUPPLEMENTARY MATERIALS

Supplementary Material 1. Transmission rate between age groups

Supplementary Material 2-1. Extended model simulation results when 60% of population except age under 18 (31,021,260) is vaccinated. (A) Daily case number, (B) Cumulative case number, (C) Additional mortality number, (D, E) Effective reproductive number (asterisk marker indicates the timing that reproductive number is consistently below 1). Note that text on the figure in panel (E) indicates which age group is vaccinated in each phase. Supplementary Material 2-2. Extended model simulation results when 80% of population except age under 18 (41,361,680) is vaccinated. (A) Daily case number, (B) Cumulative case number, (C) Additional mortality number, (D, E) Effective reproductive number (asterisk marker indicates the timing that reproductive number is consistently below 1). Note that text on the figure in panel (E) indicates which age group is vaccinated in each phase.

Korean version is available at https://www.e-epih.org/.
