# Supplementary material for: Risk of COVID-19 transmission in heterogeneous age groups and effective vaccination strategy in Korea: a mathematical modeling study
Source: Epidemiol Health. 2021 Sep 8;43:e2021059. doi: 10.4178/epih.e2021059 (PMC8769805; doi:10.4178/epih.e2021059)
Supplement: Supplementary Material 1. — Transmission rate between age groups [file epih-43-e2021059-suppl1.docx]

| $\beta_{XY}$ | Ⅰ: 0 - 17 | Ⅱ: 18 - 29 | Ⅲ: 30 - 59 | Ⅳ: 60 - 74 | Ⅴ: ≥75 |
| --- | --- | --- | --- | --- | --- |
| Ⅰ: 0 - 17 | 0.588507 | 0.157453 | 0.072937 | 0.075952 | 0.000649 |
| Ⅱ: 18 - 29 | 0.157453 | 0.785752 | 0.109814 | 0.002716 | 0.00071 |
| Ⅲ: 30 - 59 | 0.072937 | 0.109814 | 0.251084 | 0.214209 | 0.065114 |
| Ⅳ: 60 - 74 | 0.075952 | 0.002716 | 0.214209 | 0.277523 | 0.604837 |
| Ⅴ: ≥75 | 0.000649 | 0.00071 | 0.065114 | 0.604837 | 0.778924 |

**Supplementary Material 1. Transmission rate between age groups**
