# Supplementary material for: Risk of COVID-19 transmission in heterogeneous age groups and effective vaccination strategy in Korea: a mathematical modeling study
Source: Epidemiol Health. 2021 Sep 8;43:e2021059. doi: 10.4178/epih.e2021059 (PMC8769805; doi:10.4178/epih.e2021059)
Supplement: Supplementary file 2 — Supplementary Material 2-1. Extended model simulation results when 60% of population except age under 18 (31,021,260) is vaccinated. (A) Daily case number, (B) Cumulative case number, (C) Additional mortality number, (D, E) Effective reproductive number (asterisk marker indicates the timing that reproductive number is consistently below 1). Note that text on the figure in panel (E) indicates which age group is vaccinated in each phase. Supplementary Material 2-2. Extended model simulation results when 80% of population except age under 18 (41,361,680) is vaccinated. (A) Daily case number, (B) Cumulative case number, (C) Additional mortality number, (D, E) Effective reproductive number (asterisk marker indicates the timing that reproductive number is consistently below 1). Note that text on the figure in panel (E) indicates which age group is vaccinated in each phase. [file epih-43-e2021059-suppl2.docx]

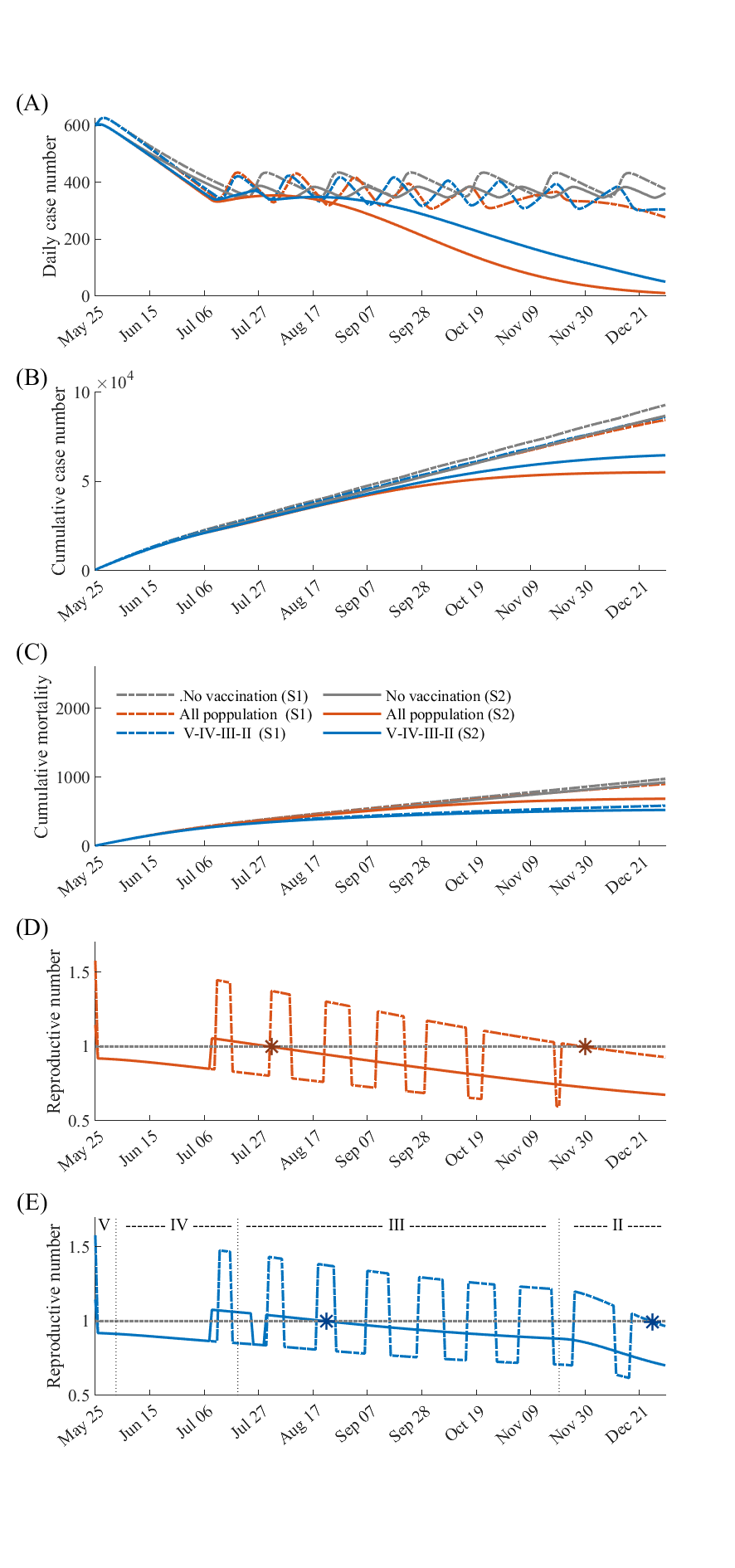


**Supplementary Material 2-1. Extended model simulation results when 60% of population except age under 18 (31,021,260) is vaccinated. (A) Daily case number, (B) Cumulative case number, (C) Additional mortality number, (D, E) Effective reproductive number (asterisk marker indicates the timing that reproductive number is consistently below 1). Note that text on the figure in panel (E) indicates which age group is vaccinated in each phase.**


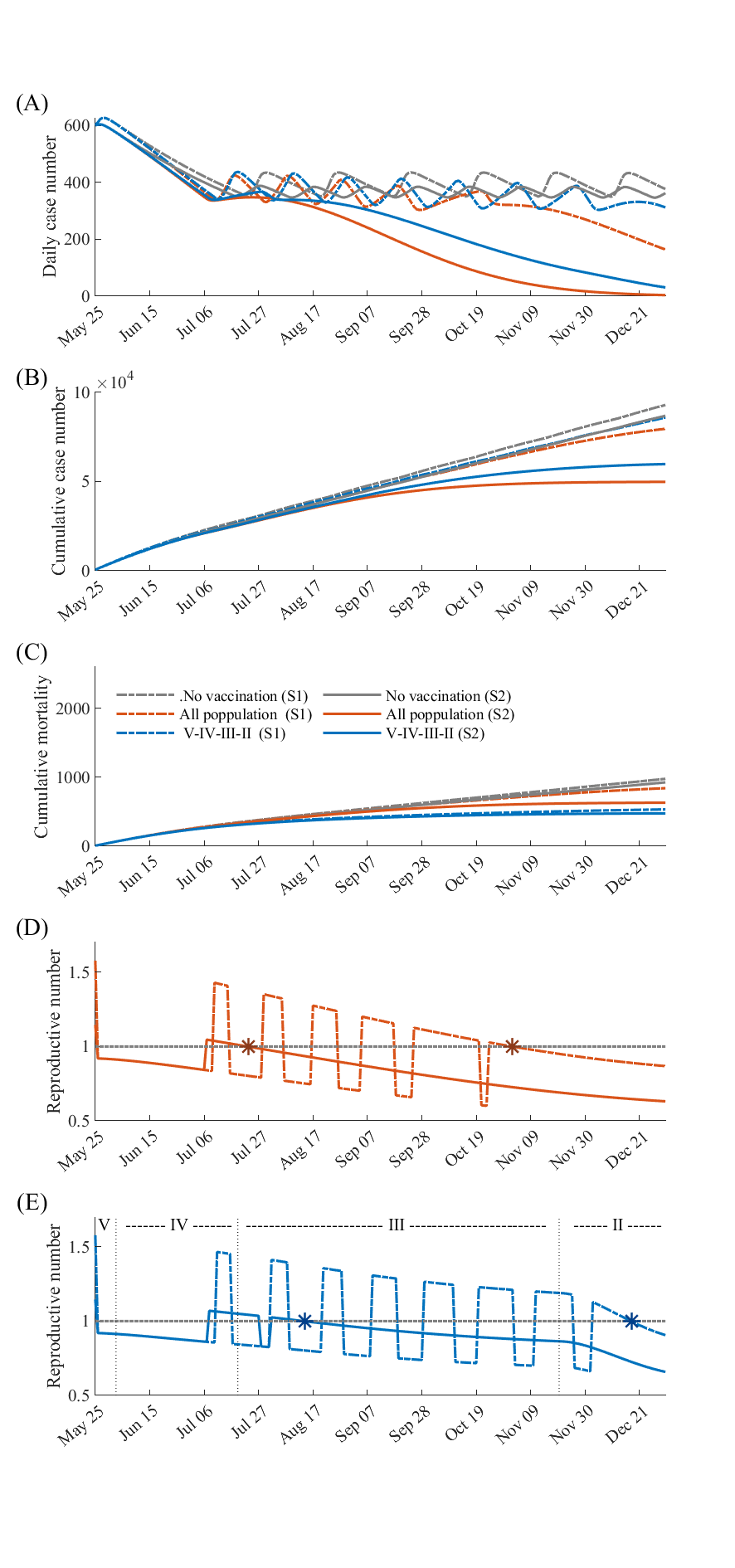


**Supplementary Material 2-2. Extended model simulation results when 80% of population except age under 18 (41,361,680) is vaccinated. (A) Daily case number, (B) Cumulative case number, (C) Additional mortality number, (D, E) Effective reproductive number (asterisk marker indicates the timing that reproductive number is consistently below 1). Note that text on the figure in panel (E) indicates which age group is vaccinated in each phase.**
