# Supplementary material for: Risk of COVID-19 transmission in heterogeneous age groups and effective vaccination strategy in Korea: a mathematical modeling study
Source: Epidemiol Health. 2021 Sep 8;43:e2021059. doi: 10.4178/epih.e2021059 (PMC8769805; doi:10.4178/epih.e2021059)
Supplement: Supplementary file 3 [file epih-43-e2021059-suppl.docx]

**Risk of COVID-19 transmission in heterogeneous age groups and effective vaccination strategy in Korea: a mathematical modeling study**

**Youngsuk Ko^(1)^, Jacob Lee^(2)^, Yubin Seo^(2)^, Eunok Jung^(1)*^**

**(1) Department of Mathematics, Konkuk University**

**(2) {Division of Infectious Disease, Department of Internal Medicine, Kangnam Sacred Heart Hospital, Hallym University College of Medicine**

*** Corresponding author, junge@konkuk.ac.kr**

Running title

A mathematical modeling study of COVID-19 epidemic

Author information

Youngsuk Ko

Department of Mathematics, Konkuk University, Seoul, Republic of Korea

E-mail: [kys1992@konkuk.ac.kr](mailto:kys1992@konkuk.ac.kr)

ORCID: 0000-0001-9063-176X

Jacob Lee

Division of Infectious Disease, Department of Internal Medicine, Kangnam Sacred Heart Hospital, Hallym University College of Medicine, KOREA , Seoul, Republic of Korea

E-mail: [litjacob@chol.com](mailto:litjacob@chol.com)

ORCID: 0000-0002-7041-065X

Yubin Seo

Division of Infectious Disease, Department of Internal Medicine, Kangnam Sacred Heart Hospital, Hallym University College of Medicine, KOREA , Seoul, Republic of Korea

E-mail: yubinseo@gmail.com

ORCID: 0000-0001-5183-1996

Eunok Jung

Department of Mathematics, Konkuk University, Seoul, Republic of Korea

E-mail: junge@konkuk.ac.kr

ORCID: 0000-0002-7411-3134

Abstract

목적(Objectives):

2020년 전세계적 코로나19 유행이 시작된 이후 여러 국가들에서 백신이 개발되고 도입되었다. 대한민국에서는 2021년 2월 26일부터 백신접종이 시작되었으며, 2021년 집단면역 형성이 목표로 설정된 상황이다. 본 연구에서는 백신 접종과 사회적 거리두기 상황을 동시에 고려하여 지속적인 감염재생산지수 1 미만 유지 가능성 및 조건을 수리모델링 기법을 이용하여 분석하고자 한다.

방법(Methods):

전체 인구집단을 다섯 개의 연령군(0-17세, 18-29세, 30-59세, 60-74세, 75세 이상)으로 나누었다. 각 연령군 간의 감염전파율을 추정하기 위하여 Maximum likelihood estimation(MLE)을 이용하였고, 추정된 감염전파율 행렬을 수리모델링에 적용하였다. 수리모델은 백신 접종을 추가적으로 고려하였으며 2021년 5월 25일까지 실제 현상을 반영하여 시뮬레이션 되었으며 백신 접종과 사회적 거리두기 전략을 다양하게 설정하여 2021년 12월 31일까지 변이 바이러스는 고려하지 않고 연장된 백신전략 시뮬레이션을 진행하였다.

결과(Results):

MLE 결과, 0세부터 17세 집단은 다른 연령군 대비 낮은 감염전파 위험과 고연령 집단의 상대적으로 높은 감염 위험을 관찰하였다. 2021년 말까지 국민의 70% 백신전략 시뮬레이션은 사회적 거리두기가 완화되어도 3차 재확산 시기 수준이 아닐 경우 2021년 8월을 근처로 지속적인 감염재생산지수 1 미만 유지가 됨을 보였다. 하지만 사회적 거리두기가 완화 상황이 3차 재확산 시기 수준이 될 경우에는 고연령자 우선 접종 상황에서는 2021년 말에 지속적인 감염재생산지수 1 미만 유지를 이루어질 수 있음을 관찰하였다.

결론(Conclusions):

수리모델 시뮬레이션 결과는 안정적인 감염병 통제 상황과 악화된 상황을 모두 보여주었다. 악화된 상황의 시뮬레이션은 사망자 최소화 전략 및 2021년의 지속적인 감염재생산지수 1 미만 유지를 동시에 효과적으로 달성하기 위해서는 충분한 백신 공급과 더불어 재확산 시기의 수준의 유행이 다시 발생하지 않도록 사회적 거리두기의 유지를 통한 유행 상황의 통제가 필요함을 보여주었다.

중심단어(Key words)

코로나바이러스감염증19(COVID-19), 수리모델(Mathematical model), 최대우도추정법(Maximum likelihood estimation), 백신 전략(Vaccination strategy), 사회적 거리두기(Social distancing), 대한민국(Republic of Korea)

**List of English abbreviation**

COVID-19: Coronavirus disease 19

KCDA: Korea Disease Control and Prevention Agency

MLE: Maximum likelihood estimation

S: Susceptible

E: Exposed

I: Infectious

Q: Quarantine

R: Recovered

U: Unprotected

V: Vaccinated

P: Protected

Introduction

2019년 말 중국에서의 유행을 시작으로 전 세계적으로 확산된 코로나바이러스감염증-19(COVID-19)는 현재인 2021년 5월 25일까지 총 1억 6000여만명의 확진자와 3백만 명의 사망자, 일일 60여만 명의 신규 확진자가 보고되고 있으며, 높은 전파력과 고연령군의 높은 중증도는 전세계적으로 의료시스템에 부담을 주고 있고 봉쇄와 같은 비약물적 중재는 사회/경제적 부담을 야기하고 있다[1]. 상황의 심각성으로 인하여 COVD-19 백신 개발은 긴급하게 진행되었고 AZD1222 백신이 2020년 12월 영국에서 최초로 긴급사용승인을 받은 후 접종이 시작되었으며 BNT162와 같은 백신들이 여러 국가들에서 추가적으로 사용승인 및 접종되고 있는 상황이다[1, 2].

대한민국은 2020년 2원 16일 지역사회 감염 확산 첫 확진자가 발생하여, 종교집단 중심 집단감염으로 대구/경북에서 1차 유행이 시작되었고, 2020년 2월 29일 최대 일일 신규 확진자수 909명이 발생하였다. 그 이후 수도권 중심 2차 유행(최대 신규 확진자수 441명, 2020년 8월 27일)과 전국 단위의 산발적인 감염으로 진행된 3차 유행(최대 신규 확진자수 1237명, 2020년 12월 25일)을 겪었다[3, 4 ,5]. 3차 유행의 최대 확진자 발생 이후 사회적 거리두기의 강화된 형태로서 수도권에선 5인 이상의 사적 모임을 금지하는 정책이 시행되었으며 이후 확진자수는 500명대 아래까지 감소하였다.

대한민국의 백신 접종은 2021년 2월 26월 최초로 시작하여 5월 25일까지 총 3,864,784명에 대해 한 번 이상의 접종이 된 상황이다[6]. 한국 정부는 2021년 말까지 총 1억 9천만 회 분 백신을 도입할 예정이며 중증환자와 사망자 수 최소화를 위하여 요양병원과 요양시설 입소자 및 종사자와 고위험 의료기관 종사자 등을 우선으로 시작으로 2분기에는 고연령자에 대한 백신접종이 예정되어 있다[7, 8]. 질병관리청 5월 6일자 보도자료에 따르면 백신의 효과는 60세 이상 대상으로 1차 접종 이후 2주 후 AZD1222는 86%, BNT162는 89.7%로 분석되었으며 이상 반응 신고율은 0.1% 수준이었다[8].

수리모델링 기법을 이용한 COVID-19 확산 연구는 한국에서의 유행이 발생하기 직전부터 진행되었다. 2020년 초반 중국에서만 유행하던 COVID-19가 한국에 유입될 경우 발생할 수 있는 규모에 대한 연구를 시작으로 초기 유행 상황을 인구집단의 행동의 변화를 고려하여 해석한 연구가 진행되었으며, 1차 유행 이후 관찰된 이동량 데이터를 활용한 거리두기 효과 분석 연구가 진행되었다[9, 10, 11].

본 연구에서는 선행연구들을 발전시켜 연령군과 백신 접종을 동시에 고려한 수리모델을 개발하였다. 수리모델 시뮬레이션 기법을 통하여 5월 25일까지의 코로나19 유행이 구현되었으며 이후 2021년의 백신접종계획을 고려하여 연장된 시뮬레이션이 진행되었다. 사회적 거리두기, 백신접종량, 그리고 백신접종 방법에 따라 고안된 다양한 시나리오 분석을 통하여 본 연구에서는 지속적인 감염재생산지수 1 미만 유지, 그리고 사망자 수 최소화 전략의 동시 달성 가능성에 대해 토의하고자 한다.

Materials and methods

Maximum likelihood estimation (MLE)을 이용한 연령군 간의 감염전파율 행렬 추정

연령군별 감염전파 행렬의 추정은 최대우도추정(MLE) 방법을 사용하여 질병관리청에서 제공된 2020년 10월부터 2021년 2월 14일까지 보고된 개별 확진자 데이터 중 연령, 증상 유무, 증상 발현일, 그리고 진단일이 사용되었다. 전체 인구는 백신 정책과 연령군별 행동 이질성을 고려하여 다섯 개로 나누어졌으며 각 연령대 및 인구수는 다음과 같다[12].

Ⅰ: 0세부터 17세(7,625,814명)

Ⅱ: 18세부터 29세(7,781,166명)

Ⅲ: 30세부터 59세(23,650,843명)

Ⅳ: 60세부터 74세(9,020,169명)

Ⅴ: 75세 이상(3,624,108명)

연령군 간의 감염전파율 행렬은 5×5 행렬이 된다. 본 연구에서 감염전파율 행렬은 대칭이라 가정하였으며, 추정되는 감염전파율의 수는 총 15개이다. MLE를 설정하기 위해 연령군 $Y$의 개인이 연령군 $X$의 개인을 감염시킬 전파율을 $\beta_{XY}$라 할 경우, 감염이라는 사건이 지역사회에서 감염자와 피감염자의 접촉이 균일(homogeneous mixing)하게 있다 가정하고 감염이라는 사건은 exponential distribution을 따른다 하면 시점 $t$에서 연령군 $X$의 개인 $i$가 시점 $t+1$에서도 감염되어있지 않을 확률은 $p_{sur,X, i}\left( t \right)=exp(-\frac{\sum\beta_{XY}I_{Y}(t)}{N})$이다[13]. 여기서 $I_{Y}(t)$는 시점 $t$에서 감염전파를 시킬 수 있는 연령군 $Y$의 감염자 수고 $N$은 전체 인구 수인데, 격리와 사망자 수는 그 수의 합이 최대였던 순간이 10,000명을 상회하는 수준이었고 전체 인구가 50,000,000명 수준인 것에 비해서 0.02%로 작기 때문에 고정된 인구 수로 반영하였다. 반대로 $t+1$에 감염되어있을 확률은 $p_{inf,X,i}\left( t \right)=1-exp(-\frac{\sum\beta_{XY}I_{Y}(t)}{N})$라 할 수 있다. 피감염과 감염될 확률을 이용하여 모든 개인들(마지막 순간까지 감염되지 않은 개인 포함)의 우도를 설정할 수 있으며 이는 다음과 같다.

$$L=\prod_{X} \left\{ \prod_{i\in\Lambda_{I, X}} \left[ \left( \prod_{j=0}^{t_{inf, x, i}-2} p_{sur,X,j}\left( t \right) \right)p_{inf, X, i}\left( t_{inf,X,i}-1 \right) \right]\prod_{k\in\Lambda_{S, X}} \left( \prod_{j=0}^{t_{f}-1} p_{sur, X,j}\left( t \right) \right) \right\}$$

개별 환자들이 실제로 감염된 날짜에 대한 정보가 없으므로 증상이 있는 감염자는 증상 발현 4일 전에 감염되었고, 증상 발현 2일 전부터 진단되기 하루 전까지 감염전파를 시킨다고 설정하였다[14, 15]. 무증상 감염자의 경우는 증상이 있는 감염자들의 평균적인 수치를 반영하여 동일한 방법으로 감염된 날짜와 감염전파력을 지니게 된 날짜를 설정하였다.

코로나19 유행에 대한 수리모델링

앞서 고려된 5개의 연령군을 고려하여 백신 접종이 반영된 수리모델이 개발되었다. 전체 인구는 COVID-19의 역학적 특성에 따라 감수성(Susceptible$: S$), 잠복기(Latent or Exposed$: E$), 감염노출 (Infectious: $I$), 확진 후 병원격리(isolated$: Q$), 회복 혹은 사망(Recovered: $R$) 그룹으로 나누어진다. 백신 접종을 반영하기 위해 세 개 그룹($U$, $V$, $P$)을 추가적으로 고려하였다: 백신을 접종했지만 효과를 보지 못한 그룹(Unprotected: $U$), 효과적으로 백신에 접종되었지만 아직 면역이 형성되지 않은 그룹(Vaccinated: $V$), 그리고 효과적으로 백신 접종이 된 후 일정 시간을 거쳐 면역형성이 된 그룹(Protected$: P$). 따라서 감염될 수 있는 감수성 집단은 $S$, $U$, $V$ 이며 이들은 모두 동일한 감수성을 지녔다고 가정하였다. 수리모델 흐름도는 Figure.1에 표현되어 있으며 수리모델 지배방정식은 다음과 같다.

$\frac{dS_{X}}{dt}=-\Lambda_{X}S_{X}-\nu_{X},$

$\frac{{dE}_{X}}{dt}=\Lambda_{X}\left( S_{X}+U_{X}+V_{X} \right)-\kappa E_{X},$

$\frac{{dI}_{X}}{dt}=\kappa E_{X}-\alpha I_{X},$

$\frac{{dQ}_{X}}{dt}=\alpha I_{X}-\gamma Q_{X},$

$\frac{dR_{X}}{dt}=\gamma(1-f_{X})Q_{X},$

$\frac{dU_{X}}{dt}=(1-p)\nu_{X}-\Lambda_{X}U_{X},$

$\frac{dV_{X}}{dt}=p\nu_{X}-\Lambda_{X}V_{X}-\omega V_{X},$

$\frac{dP_{X}}{dt}=\omega V_{X},$

$\Lambda_{X}=\beta_{0}(t)\frac{\sum\beta_{XY}I_{Y}}{N},$ $N=\sum S_{X}+E_{X}+I_{X}+R_{X}+U_{X}+V_{X}+P_{X}.$

수리모델에서 각 연령군은 아래 첨자를 이용하여 표기되었으며 MLE를 이용한 연령군 간의 감염전파율은 $\beta_{XY}$로 적용되었으며 이는 3차 유행기간 동안의 평균적인 전파율이므로 시기마다 변화하는 방역정책과 전파 양상을 고려하여 보정상수 $\beta_{0}(t)$가 추가적으로 감염 항에 적용되었다. 방역정책과 전파 양상에 따라 유행 트랜드는 바뀌지만, 전 시뮬레이션 기간동안 연령군들 사이의 감염전파 패턴은 항상 동일하게 적용된다고 가정하였다. 백신 접종은 $\nu_{X}$을 이용하여 모델에 반영되었으며 $\beta_{0}(t)$ 추정 시뮬레이션 기간까지는 실제 접종량이 반영되었다[6]. $\beta_{0}(t)$는 수리모델에서 계산되는 전체 인구집단의 누적 확진자수($\int\sum\alpha I_{X}dt$)와 검역을 제외한 질병관리청의 COVID-19 전국 누적 확진자수 보도자료 데이터와의 제곱오차를 최소화하는 방법으로 추정되었다. 추정 기간은 대한민국 3차 유행 시작 전 사회적 거리두기 1단계 시작인 2020년 10월 12일부터 2021년 5월 25일로 설정되었다[16]. 모수 $p$는 평균적인 백신 효과를 나타내며, 실제 우리나라에서 접종된 백신 종류별 접종량과 백신 효과를 가중평균으로 계산하여 0.84로 설정되었다[17]. 모델 모수들의 값은 Table.1에 나열되어 있으며, 연령군별 사망률은 개별 확진자 데이터 중 격리 해제자들을 기준으로 계산되었다. 본 연구에서 감염재생산지수(effective reproductive number: $R\left( t \right)$)는 5개 연령군과 각 연령군 안에 8개 역학그룹 구조로 인해 복잡한 식으로 표현되므로 결과 세션에서 next-generation 방법을 사용하여 계산된 감염재생산지수를 수치로 시각화 하였다[18].

Figure.1 Flowchart of COVID-19 mathematical model considering age group and vaccination

**Table.1 Model parameters**

2021년 백신 접종 시나리오

백신 접종에 따른 COVID-19 유행 상황을 예측하기 위하여 보건복지부의 사회적 거리두기 체계 개편안의 단계 전환 기준을 참고하여 시나리오를 설정하였다[21]. 개편안에 의해 권역 중환자실의 여유를 고려한 인구 10만명당 일주일 평균 일일 신규 확진자 수를 기준으로 거리두기 단계가 전환되는데, 전국 단위에서는 363명이 1단계와 2단계의 전환 기준점이고 778명은 2단계와 3단계, 1556명은 3단계와 4단계의 전환 기준이다. 주간 평균 600명 아래인 2021년 5월 25일 기준 유행추세를 고려하여 3단계는 설정하지 않았으며 2021년 하반기 기간동안 변이 바이러스의 출현, 재확산 혹은 코로나 유행의 종식 없이 1단계와 2단계가 지속적으로 반복될 것을 가정하여 시나리오를 설정하였다. 1단계와 2단계 상황일때의 유행 상황은 3차 유행 기간동안 추정된 감염전파 행렬과 보정상수 $\beta_{0}(t)$를 이용하여 다음 두 가지 시나리오를 설정하였다:

- 시나리오1(**S1**): 2단계는 3차 유행 당시 $\beta_{0}(t)$가 가장 작은 값 적용(당시 $R\left( t \right)=0.71$ , 1단계는 3차 유행 중 가장 큰 값(당시 $R\left( t \right)=1.68)$
- 시나리오2(**S2**): 2단계는 3차 유행 당시 $\beta_{0}(t)$가 가장 작은 값 적용(당시 $R\left( t \right)=0.71$ ), 1단계는 3차 유행 직전의 1단계 상황(당시 $R\left( t \right)=1.22$, 2020년 10월 12일 - 2020년 11월 3일)

백신 접종 방법은 세가지를 고려하였다; 고연령군 우선 접종(그룹 Ⅴ-Ⅳ-Ⅲ-Ⅱ의 순서), 17세 이하(Ⅰ)를 제외한 전체 연령군에 인구 대비 동일한 비율로 동시 접종, 그리고 백신 접종이 없는 경우. 고연령군 우선 접종 방법에서는 고연령 순서대로 각 연령군이 순서대로 접종을 마치면 다음 연령군이 접종되는 방법으로 백신 접종이 적용되었다. 두 접종 상황 모두 연령군 Ⅰ는 접종되지 않는다. 즉, 전체 인구의 60%(70, 80%)가 접종이 될 경우 연령군 Ⅰ를 제외한 나머지 연령군들은 각각 약 70%(82, 94%)가 접종을 받게 된다. 백신 총 접종량은 상황에 따라 유동적으로 변할 수 있으므로 2021년 12월 31일까지 한국 전체 인구의 60, 70, 80%(31,021,260, 36,191,470, 41,361,680)를 접종하는 상황을 설정하였다. 이에 따라 설정되는 시나리오의 수는 총 14개이다. 시뮬레이션은 모수 추정의 가장 마지막 날인 2021년 5월 25일부터 2021년 12월 31일까지 연장되어 진행되며, 일일 백신 접종량은 설정된 총 백신 접종량에 맞춰 평균적으로 동일하게 적용된다. 관찰하는 수치는 2021년 5월 25일 이후 추가적으로 발생되는 일일 신규 확진자 수, 누적 확진자 수 및 사망자 수, 감염재생산지수, 그리고 지속적으로 감염재생산지수가 1보다 작아지는(거리두기가 완화되더라도 감염재생산지수가 1보다 작아지는) 시점이다.

Results

MLE를 이용한 연령군 간의 감염전파율 행렬 추정

추정된 연령군 간의 감염전파율($\beta_{XY}$)은 Figure.2에 시각화 되어있다. (A)는 log-scaled 된 행렬의 값이며, (B)는 각 연령군의 인구수로 보정하여 감염 위험을 계산한 결과이고 감염 위험의 요인은 연령군별로 나누어 표현되었다. 행렬의 각 성분 값들은 supplementary material 1에 나열되어 있다. 감염전파율은 대각 성분이 높게 추정되었으며 18세에서 29세 연령군 사이(Ⅱ)의 값이 0.7858로 가장 높게 추정되었고 75세 이상 연령군(Ⅴ) 간의 값이 0.7789로 두 번째로 높게 추정되었다.

Figure.2 (B)는 보정된 인구수를 사용한 그룹 별 감염 노출 위험율 그래프이다. 연령군 간 감염전파율 행렬에서 각 열에 해당하는 연령군의 인구수를 전체 인구로 나눈 값을 열 성분 합에 곱하는 방법으로 보정하여 연령군 별로 상대적으로 다른 그룹에 의한 감염에 노출될 위험을 관찰하였다. 상대적 피감염 위험율은 Ⅳ-Ⅱ-Ⅴ-Ⅲ-Ⅰ 순서로 높게 계산되었다. 60세부터 74세 연령군(Ⅳ)은 다른 집단에 의해 감염될 위험이 0.15로 가장 높았으며 그 중 30세 이상 59세 이하 연령군(Ⅲ)에 의한 감염 위험이 0.09로 절반 이상을 차지했다. 0세부터 59세까지 연령군들(Ⅰ, Ⅱ, Ⅲ)은 다른 집단에 의한 감염위험이 0.07로 비슷한 수치로 가장 낮은 수준이었다. 동일 연령군간 감염위험은 18세 이상 29세 이하 연령군(Ⅱ)이 0.12로 가장 높았으며, 60세부터 74세 연령군(Ⅳ)이 0.05로 가장 낮았다.

**Figure.2 Maximum likelihood estimation results. (A) Estimated transmission rates (log-scaled) (B) Population-adjusted risk of being infected, which is calculated by taking elementwise product of row-vectored population size of age groups and transmission matrix and dividing all entries by total population size.**

수리모델링 기반 감염재생산지수와 연령군 별 확진자 비율 분석

Figure.3는 정부 방역정책에 따른 보정상수 $\beta_{0}(t)$의 추정 결과와 이를 사용한 수리모델 결과 그래프를 보여준다. (A)는 감염재생산지수, (B)와 (C)는 각각 전체 연령군에 대한 일일 신규 확진자수와 누적 확진자수에 대한 수리모델 시뮬레이션 결과(검정색 그래프)와 질병관리청 보도자료 데이터(빨간색 네모)를 나타내다. 개별 확진자 데이터를 이용한 연령군 별 확진자수와 모델 시뮬레이션 결과를 비교한 그래프는 (D)와 (E)에서 보여주고 있다.

보정상수 $\beta_{0}(t)$는 모델의 시작 시기이자 사회적 거리두기 1단계가 시행되었던 10월 12일부터 11월 3일간은 1.09(감염재생산지수 1.22)로 추정되었으며, 11월 4일부터 11월 24일 사이에는 1.50으로(감염재생산지수 1.68) 가장 높게 추정되었다. 사회적 거리두기 단계가 2.5단계로 강화된 후 5인 이상 사적 모임 금지가 수도권에서 시작된 시기인 12월 23일부터 1월 18일 사이에는 0.64(감염재생산지수 0.71)로 가장 작게 추정되었다.

Figure.4는 수리모델 시뮬레이션 결과를 마지막 날짜인 2021년 5월 25일을 기준으로 각 연령군별로 분리하여 관찰한 결과이다. (A)에서 전체 확진자 수 중 인구의 30세부터 59세 연령군(Ⅲ)이 가장 많은 수(51,331)를 차지하는 것을 관찰할 수 있다. (B)는 각 인구집단의 인구수로 보정되어 10만명당 확진자 수를 표기한다. 0세부터 17세 연령군(Ⅰ)이 다른 연령군 대비 적은 것(157.7)을 관찰할 수 있었으며 나머지 연령군은 200명대 초반이고 그 중 60세부터 74세 연령군(Ⅳ)이 241.1로 가장 높게 나타났다.

**Figure.3 Data-fitting results: (A) Effective reproductive number, (B) Daily cases (all age), (C) Cumulative cases (all age), (D) Daily cases of the five age groups, (E) Cumulative cases of the five age groups. In (B) and (C), the red squares depict data aggregated from the KDCA daily presses and the black solid curves are data-fitted model simulation result in all age. In (D) and (E), the colored squares depict data aggregated from the epidemiological records of individual COVID-19 cases and the colored solid curves are model simulation results of the five age groups.**

**Figure.4 Model results in May-25-2021: (A) Confirmed case number of each age group (B) Case number per 100,000 of each age group.**

2021년 백신 접종 시나리오 시뮬레이션 결과

Figure.5는 전체 인구 중 70%(일일 약 14만 명 접종)를 2021년 12월 31일까지 접종할 경우의 각 시나리오 별 시뮬레이션 결과를 나타낸다. 그래프의 색은 백신 접종 방법을 표기한다. 회색은 백신이 더 이상 없을 경우, 붉은 색은 모든 연령군에 같은 비율로 백신을 접종할 경우, 푸른색은 고연령군 우선으로 백신을 접종할 경우를 나타낸다. 실선과 점선은 각 백신 접종 방법에 따른 시나리오에서 거리두기 완화 상황에서의 $\beta_{0}(t)$가 다르게 두었을 경우를 나타낸다. 점선은 시나리오1, 그리고 실선은 시나리오2의 시뮬레이션 결과이다. 지속적인 감염재생산지수 1 미만 달성 시점이 존재할 경우 별 모양 마커로 해당 시기가 표기된다. 전체 인구 중 60%, 80%에 백신을 접종하는 경우 및 백신 효과를 65%, 70%, 75%, 80%, 90%로 다양하게 설정한 결과는 supplementary material 2에 정리되어 있다.

각 시나리오별 지속적인 감염재생산지수 1 미만 달성 시점, 추가 확진자 수, 추가 사망자 수는 Table.2에 정리되어 있다. 17세 이하 연령군을 제외한 전체 인구에 동일하게 백신을 접종하는 시나리오가 고연령자 우선 접종 시나리오보다 확진자 수가 적은 것을 관찰할 수 있었다. 전체 인구의 80%를 동일한 비율로 접종할 경우, 추가 확진자 수가 44,859명으로 모든 시나리오에서 가장 작았다. 고연령자 우선 접종 시나리오들 중 가장 사망자가 많았던 경우(588)을 제외한 모든 시나리오들에서 전체 인구 동일 비율 접종 시나리오들 중 가장 사망자가 적었던 경우(581)보다 사망자 수가 적은 것을 확인할 수 있었다. 지속적인 감염재생산지수 1 미만 달성 시점은 백신 접종율이 높아질수록 앞당겨지며 전체 인구에 동일 비율로 접종을 할 경우가 더 빠름을 관찰할 수 있었다. 시나리오2처럼 사회적 거리두기가 완화되어도 재확산 시기 수준이 되지 않는 상황에서는 전체 인구 동일하게 백신 접종을 하는 경우는7월 중순(Figure 5(D) 실선) 혹은 고연령자 우선 접종을 하는 경우는 8월 중순 정도(Figure 5(E) 실선)에도 지속적인 감염재생산지수 1 미만 달성 시점이 시작될 수 있음을 시뮬레이션 결과에서 볼 수 있다.

**Figure.5 Extended model simulation results when the 70% of population except age under 18 (36,191,470) is vaccinated. (A) and (B) display the number of daily and cumulative cases, respectively. (C) shows the number of additional mortalities. (D) and (E) represents the effective reproductive number for Scenario 1 and 2, respectively (the asterisk marker indicates the timing that reproductive number is consistently below 1). Note that text on the figure in panel (E) indicates which age group is vaccinated in each phase.**

**Table.2 Extended model simulation results considering various scenarios**

Discussions

MLE를 이용한 연령군 간의 감염전파율 추정은 18세부터 29세 연령군 간의 높은 감염전파율과 60세 이상 고연령군에서의 높은 감염전파율을 보여주었다(Figure.2). 이들의 인구수로 보정된 수리모델 결과는 3차 유행 상황에서의 확산 위험이 특정 연령군에만 국한되지 않고 전체적으로 비슷한 수준임을 보여주었다. 0세부터 17세의 경우는 다른 연령군 대비 상대적으로 낮은 감염전파율을 보였는데 이는 사회적 거리두기의 일환인 휴교, 개학연기, 온라인수업 진행에 의한 효과 뿐 아니라 소아와 청소년의 바이러스 전파율이 성인에 비해 낮다는 바이러스 자체의 특성에 의한 것으로 보인다[22]. 이는 학동기 연령군이 나머지 연령군 대비 감염전파율이 약 9배 적게 추정되었던 선행연구 결과에서도 관찰되었다[23].

2021년 5월 25일부터 12월 31일까지 연장된 수리모델 시뮬레이션 결과는 효과적인 백신 전략을 위해서는 사회적 거리두기 단계 유지가 중요함을 보여준다. 각자 다른 백신 접종 방법(전체 연령군 동시 접종, 고연령 우선 접종)에 거리두기 상황을 다르게 고려하여 설정된 두 시나리오(**S1**, **S2**)들은 거리두기가 완화될 경우 감염전파율을 재확산 상황 수준으로 높아질지(**S1**, 당시 $R\left( t \right)=1.68$), 혹은 실제 10월 12일 시행된 거리두기 1단계 상황에서의 초기 수준으로 **S1** 대비 낮을지(**S2**, 당시 $R\left( t \right)=1.22$)를 고려하여 설정된 시나리오이다. **S1**일 경우, 현행 계획을 반영한 고연령자 우선 백신 접종 시나리오에선 전 국민의 70%가 2021년 동안 백신에 접종되어도 12월이 넘어서야 지속적인 감염재생산지수 1 미만 유지 시작을 달성할 수 있기 때문이다. 이는 고연령자 우선 접종이 적절하지 않다는 결론은 아니다. 사망자 수는 고연령자를 우선적으로 접종할 경우, 가장 악화된 상황(백신접종률 60%, 시나리오1)에서의 588명을 제외하면 전체 인구집단에 동일한 비율로 백신을 투여할 경우의 최고의 상황(백신접종률 80%, 시나리오2)에서의 사망자 수인 581명보다 적기 때문이다(Table.2). **S2**처럼 사회적 거리두기가 재확산 상황 수준으로 돌아가지 않도록 지켜진다면, 국민의 70%를 12월 말까지 백신 접종을 시행할 경우 지속적인 감염재생산지수 1 미만 유지 시작 시점이 8월로 가능할 수도 있음을 시뮬레이션 결과에서 보여주었다(Figure.5 D와 E 실선).

본 연구에서의 한계점은 다음과 같다. 첫째, 모델의 복잡성을 줄이기 위하여 2차 백신 접종을 고려하지 않았으며 백신의 종류와 백신 효과가 평균적으로 반영되었으며 loss of immunity와 접종으로 인해 감염이 되더라도 확률이 줄어드는 등의 효과는 고려되지 않았고 연장된 시뮬레이션의 일일 백신 접종량은 동일한 수치로 반영되었다. 둘째, 연령군 간의 감염전파율은 최근으로 올수록 백신 접종 등으로 변했을 가능성이 있으며 특히 고연령자 집단에서의 감염전파율이 본 연구에서는 과다하게 추정되었을 수도 있지만, 본 연구에서는 3차 유행 기간 동안의 전체적인 감염전파 현상이 평균적으로 반영되었다. 데이터의 한계로 인하여 지역사회에서의 homogeneous mixing을 가정하여 추정된 감염전파율은 실제 감염의 인과관계를 그대로 내포하는 것이 아니므로 실제 접촉 동향과는 다를 수 있다. 셋째, 본 연구에서 변이 바이러스의 등장으로 인한 감염전파력의 증가는 반영되지 않았다. 따라서, 변이 바이러스의 등장으로 인하여 지속적인 감염재생산지수 1 미만 도달 시기는 각 시나리오에서 지연될 수 있다. 단, 본 연구결과에서 사회적 거리두기 완화 시나리오의 경우 감염전파율이 높은 값(당시 $R\left( t \right)=1.68$)은 변이 바이러스에 의한 감염전파력 증가 상황을 반영하는 것으로도 해석할 수도 있다. 2차 백신 접종과 유행 시기별 감염전파율, 그리고 loss of immunity와 자세한 백신 효과, 변이 바이러스를 고려한 연구는 추후에 진행될 예정이다.

Conclusions

본 연구에서 대한민국 데이터에 근거하여 MLE를 이용, 연령군 사이의 감염전파율을 추정한 후 백신 수리모델을 개발하였고, 2021년 백신 접종 계획을 고려한 시뮬레이션을 다양한 시나리오로 진행하여 감염재생산지수, 감염자 수, 그리고 사망자 수 최소화의 관점에서 결과를 분석하였다. 수리모델 시뮬레이션의 중요 결과 중 하나는 효과적인 백신전략을 위해서는 백신우선순위, 빠른 백신접종 뿐 아니라 감염재생산지수를 3차 유행 시기의 재확산 수준이 되지 않도록 하는 사회적 거리두기가 같이 병행되어야 한다는 것이다.

고연령자 우선 접종 전략이 이에 비해 확진자 수는 더 많이 줄여줄 수 있는 다른 방법보다도 사망자 수를 월등히 줄일 수 있는 방법이라는 점을 보여주며 백신 우선순위 설정의 목적과 효과가 동일함을 보여준다. 하지만 악화된 상황에서는 2021년에 유행이 지속될 것 또한 시뮬레이션 결과는 보여주고 있으며, 그러한 상황에서도 사망자 수 최소화 효과의 유효성은 변하지 않기 때문에 연구의 결과는 백신 우선순위 변경이 아닌 사회적 거리두기의 중요성을 역설한다. 2021년 12월 말까지 국민의 70% 백신 접종과 3차 유행 시기의 재확산 수준이 되지 않도록 하는 사회적 거리두기가 이루어진다면, 8월 정도부터 감염재생산지수가 지속적으로 1보다 작아질 수도 있음을 수리모델링으로 관찰하였다.

References

1. Johns Hopkins University; COVID-19 Dashboard by the Center for Systems Science and Engineering (CSSE), Available from: <https://www.arcgis.com/apps/opsdashboard/index.html#/bda7594740fd40299423467b48e9ecf6>
2. Vaccine Centre- London School of Hygiene and Tropical Medicine; COVID-19 vaccine development pipeline. Available from: <https://vac-lshtm.shinyapps.io/ncov_vaccine_landscape/>
3. Korea Disease Control and Prevention Agency; Korean situation report of COVID-19; 2020 February 29
4. Korea Disease Control and Prevention Agency; Korean situation report of COVID-19; 2020 August 27
5. Korea Disease Control and Prevention Agency; Korean situation report of COVID-19; 2020 December 25
6. Korea Disease Control and Prevention Agency; Vaccination in Korea overview, Available from: https://ncv.kdca.go.kr/mainStatus.es?mid=a11702000000
7. Korea Disease Control and Prevention Agency; COVID-19 vaccination, when and where it will be?, Available from: https://ncv.kdca.go.kr/menu.es?mid=a10117010000
8. Korea Disease Control and Prevention Agency; Appendix 1: Korean situation report of COVID-19; 2021 May 5
9. Kim S, Choi S, Ko Y, Ki M, Jung E. Risk estimation of the SARS-CoV-2 acute respiratory disease outbreak outside China. Theoretical Biology and Medical Modelling. 2020 Dec;17:1-7.
10. Kim S, Seo YB, Jung E. Prediction of COVID-19 transmission dynamics using a mathematical model considering behavior changes in Korea. Epidemiology and health. 2020;42.
11. Kim S, Kim YJ, Peck KR, Ko Y, Lee J, Jung E. Keeping Low Reproductive Number Despite the Rebound Population Mobility in Korea, a Country Never under Lockdown during the COVID-19 Pandemic. International journal of environmental research and public health. 2020 Jan;17(24):9551.
12. Ministry of the Interior and Safety; Administrated population statistics- population by age
13. Myung IJ; Tutorial on maximum likelihood estimation. Journal of mathematical Psychology. 2003 Feb 1;47(1):90-100.
14. Korea Disease Control and Prevention Agency; Korean situation report of COVID-19; 2020 Feb 16
15. World Health Organization; Transmission of SARS-CoV-2: implications for infection prevention precautions: scientific brief, 09 July 2020. World Health Organization; 2020.
16. Korea Disease Control and Prevention Agency; Korean situation report of COVID-19; 2021 May 25.
17. Park E, Lee S, Kim S, Park Y, Lee J, Lim D; COVID-19 vaccination effectiveness analysis in Korea, Korea Centers for Disease Control and Prevention, Weekly health and diseases 2021;vol14:19
18. Diekmann O, Heesterbeek JA, Roberts MG. The construction of next-generation matrices for compartmental epidemic models. Journal of the Royal Society Interface. 2010 Jun 6;7(47):873-85.
19. Ki M; Task Force for 2019-nCoV. Epidemiologic characteristics of early cases with 2019 novel coronavirus (2019-nCoV) disease in Korea. Epidemiol Health 2020;42:e2020007.
20. Lee YH, Hong CM, Kim DH, Lee TH, Lee J. Clinical course of asymptomatic and mildly symptomatic patients with coronavirus disease admitted to community treatment centers, South Korea. Emerging infectious diseases. 2020 Oct;26(10):2346.
21. Ministry of Health and Welfare of South Korea, Social distancing system reform plan, 2021 Mar.
22. Li F, Li YY, Liu MJ, Fang LQ, Dean NE, Wong GW, Yang XB, Longini I, Halloran ME, Wang HJ, Liu PL. Household transmission of SARS-CoV-2 and risk factors for susceptibility and infectivity in Wuhan: a retrospective observational study. The Lancet Infectious Diseases. 2021 May 1;21(5):617-28.
23. Kim S, Kim YJ, Peck KR, Jung E. School opening delay effect on transmission dynamics of coronavirus disease 2019 in Korea: based on mathematical modeling and simulation study. Journal of Korean medical science. 2020 Apr 6;35(13).
